# A miR-200c/141-BMI1 autoregulatory loop regulates oncogenic activity of BMI1 in cancer cells

**DOI:** 10.18632/oncotarget.8811

**Published:** 2016-04-18

**Authors:** Manjari Dimri, Mingu Kang, Goberdhan P. Dimri

**Affiliations:** ^1^ Department of Biochemistry and Molecular Medicine, School of Medicine and Health Sciences, The George Washington University, Washington, DC, USA

**Keywords:** breast cancer, senescence, BMI1, microRNA, miR-141

## Abstract

MicroRNAs (miRNAs) are known to function as oncomiRs or tumor suppressors and are important noncoding RNA regulators of oncogenesis. The *miR-200c/141* locus on chromosome 12 encodes miR-200c and miR-141, two members of the miR-200 family, which have been shown to function as tumor suppressive miRNAs by targeting multiple oncogenic factors such as polycomb group protein BMI1. Here, we show that BMI1 reciprocally functions as a transcriptional repressor of the miR-200c/141 cluster and that BMI1 inhibitors upregulate expression of miR-200c and miR-141. Our data suggest that BMI1 binds to the *miR-200c/141* promoter and regulates it through transcription factor binding motifs E-box 2 and Z-box 1 to repress expression of miR-200c/141 cluster. We also show that PTC-209, a small molecule inhibitor of *BMI1* gene expression induces cellular senescence and transcriptionally upregulates expression of miR-200c/141 cluster in breast cancer cells. Furthermore, inhibition of expression of miR-200c or miR-141 overcomes tumor suppressive effects of PTC-209 including induction of cellular senescence and downregulation of breast cancer stem cell phenotype. Therefore, our studies suggest a reciprocal regulation between BMI1 and miR-200c/141 cluster, and that BMI1 inhibitory drugs can further amplify their inhibitory effects on BMI1 via multiple mechanisms including posttranscriptional regulation by upregulating BMI1 targeting miRNAs.

## INTRODUCTION

MicroRNAs (miRNAs) are evolutionarily conserved small non-coding RNA molecules of 19-24 nucleotides in length, which control expression of target genes via base pairing to seed sequences that are found in 3′ untranslated region of a particular target gene [1, 2]. MiRNAs have recently emerged as major regulators of cancer and other human diseases [3, 4]. During cancer progression, miRNAs can function either as oncogenes (oncomiRs) or tumor suppressors [5, 6]. Accordingly, several miRNAs are known to be significantly downregulated or overexpressed in various cancer cells including breast cancer cells [7]. For example, miR-21, miR-155, miR-10b and miR-29 are overexpressed, whereas let-7, miR-200 family, miR-125a/b, miR-206, miR-17, miR-34a and miR-31 are downregulated in breast cancer cells [7, 8].

The miR-200 family members, which include miR-200c, miR-141, miR-200a/b and miR-429, exert tumor suppressor function by inhibiting epithelial to mesenchymal transition (EMT), cell motility, anoikis resistance and cancer stem cell phenotype [9–11]. The PcG proteins BMI1 and EZH2 are two important constituents of the polycomb repressive complex PRC1 and PRC2 respectively [12, 13]. The constituents of PRC1 and PRC2 including BMI1 and EZH2, are often overexpressed in breast, prostate and colon cancer cells [14–16], and regulate oncogenic phenotypes [17–20]. In particular, PcG protein BMI1 promotes cancer stem cell (CSC) phenotype and therapy resistance in cancer cells [14, 21]. It also regulates cellular senescence via repression of tumor suppressor p16INK4a [22, 23]. Because of its role in promoting oncogenic properties including CSC phenotype, inhibitors of BMI1 are of clinical importance. These inhibitors include miRNAs (miR-200c, miR-141 and miR-31), WNT inhibitors and certain pharmacological inhibitors [24–28], and a recently described cell permeable small molecule inhibitor PTC-209 [29]. PTC-209 represents a new class of inhibitors of gene expression that was identified using Gene Expression Modulation by Small Molecules (GEMS) technology designed to screen out small molecules that can inhibit *BMI1* gene expression via interaction with its 5′ and 3′ untranslated regions (UTR) [29].

The miR-31 was recently shown to be negatively regulated by the PcG protein EZH2 in adult T cell leukemia (ATL) cells [30]. In addition, we recently reported that PcG protein BMI1 is a negative regulator of miR-31 [26]. Recently, we showed that expression of the PcG proteins is inhibited by histone deacetylase inhibitors (HDACi) [24], and that HDACi may work through upregulation of miR-200c/141 cluster [27]. We also showed that inhibitors of polo-like kinase 1 (PLK1) can upregulate miR-200c/141 cluster, which indirectly results in downregulation of BMI1 and cancer stem cell phenotype [28]. In this study, we show that similar to miR-31 regulation by PcG proteins, BMI1 negatively regulates expression of miR-200c and miR-141, which targets BMI1 mRNA for degradation [27]. We further studied regulation of miR-200c/141 cluster by PTC-209, a clinically relevant small molecule inhibitor of BMI1 and CSC phenotype [29].

## RESULTS

### BMI1 transcriptionally regulates expression of *miR-200c/141* cluster

**Figure 1:**
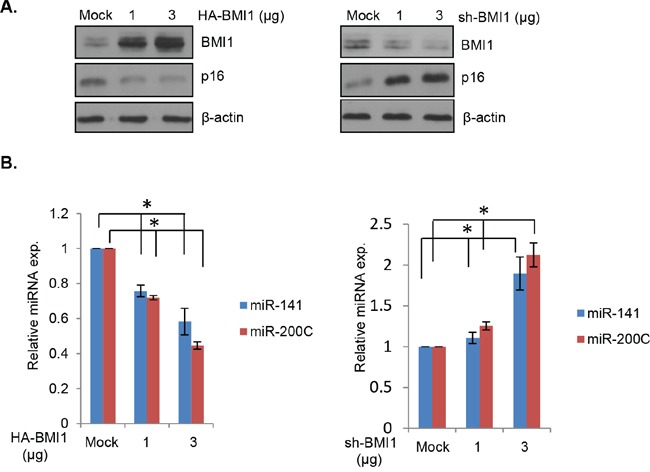
BMI1 regulates expression of miR-141 and miR-200c **A.** 293T cells were transiently transfected with HA-BMI1 (left panel) or pRS-shBMI1 (right panel). After 48 hrs, the total cell lysates were prepared from transfected cells and the expression of BMI1, p16 (a BMI1 target) and β-actin was detected by Western blot analysis using specific antibody against each protein. **B.** Downregulation of miR-141 and miR-200c by BMI1 overexpression (left panel), and upregulation of miR-141 and miR-200c by BMI1 knockdown (right panel) were confirmed by qRT-PCR in the above set of cells as described in the Methods. Error bars represent ±S.D, * *p* <0.05.

The EMT transcription factor ZEB1 negatively regulates miR-200c/141 cluster via an autoregulatory loop [31]. We recently showed that both miR-200c and miR-141 can target BMI1 [28]. We have also reported that an indirect inhibition of BMI1 by PLK1 inhibitor can lead to upregulation of miR-200c/141 cluster [28], suggesting that BMI1 may directly regulate it via an autoregulatory loop similar to the reciprocal regulation of ZEB1 and miR-200c/141 cluster. To test this hypothesis, we transiently overexpressed BMI1 or downregulated it using a transient transfection of a BMI1 shRNA vector in 293T (a derivative of HEK293) cells, and determined the expression of both miR-200c and miR-141 by qRT-PCR. The results showed that the transient BMI1 overexpression led to a dose-dependent decrease in expression of miR-200c and miR-141, and a dose-dependent increase in expression of both of these miRNAs by transient BMI1 knockdown in 293T cells (Figure 1A, 1B).

**Figure 2:**
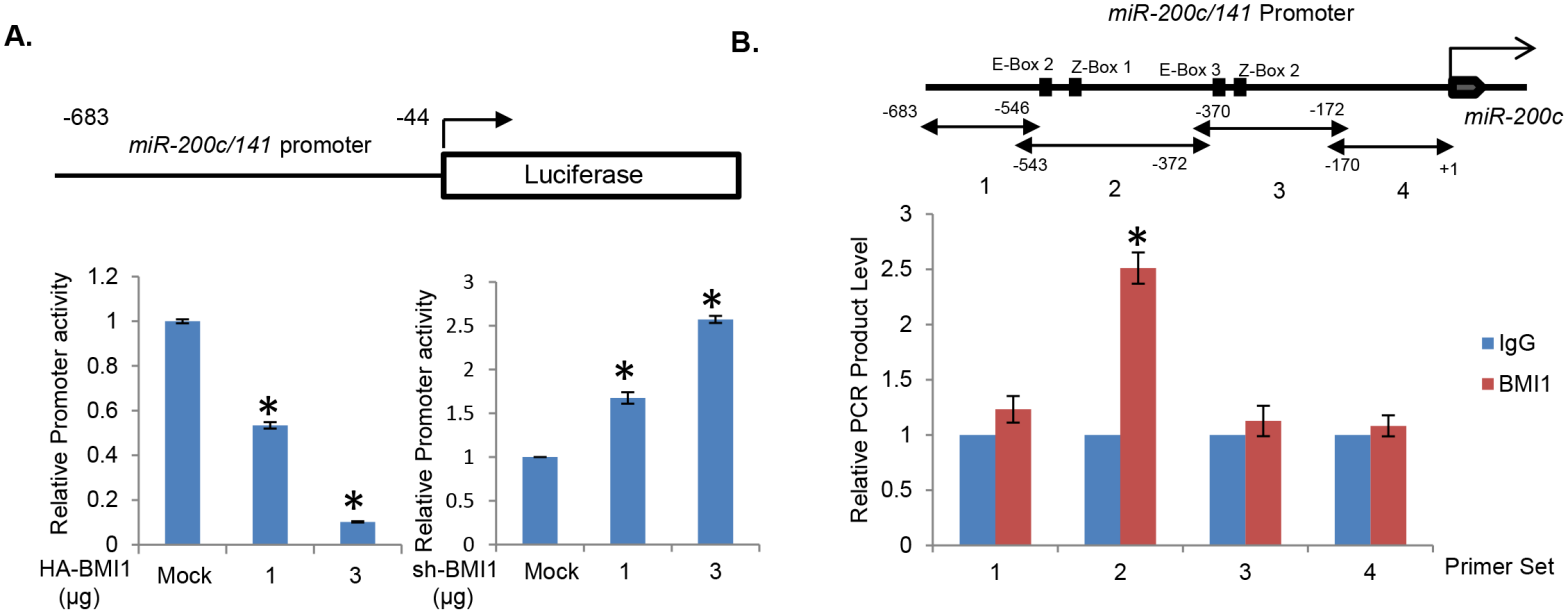
BMI1 transcriptionally regulates *miR-200c/141* promoter **A.** 293T cells were transiently transfected with miR-200c/141 promoter-reporter (pGL4.8-*PmiR-200c/141*) and pTK-Luc plasmids with HA-BMI1 or pRS-shBMI1 plasmids, and normalized luciferase activity was determined after 48 hrs and plotted. **B.** Binding of BMI1 to *miR-200c/141* promoter was studied by ChIP assay. Four different sets of primers covering 683 bp of *miR-200c/141* promoter region were designed to amplify BMI1 immunoprecipitated chromatin and the qPCR was performed as described in the Experimental Methods. The approximate position of E-box 2, Z-Box 1, E-Box 3, and Z-box 2 are indicated in the promoter region. Error bars represent ±S.D, * *p* <0.05.

To further confirm these results, and determine the mechanism of downregulation of miR-200c/141 cluster, we performed promoter-reporter assays using transient transfection of pGL-miR-200c/141 promoter construct with HA-BMI1 (for BMI1 overexpression) and pRS-BMI1shRNA (for BMI1 knockdown) plasmids in 293T cells. Our results indicated a dose-dependent decrease in the reporter activity with overexpression of BMI1 and a dose-dependent increase in its activity upon BMI1 knockdown (Figure 2A), thereby confirming transcriptional downregulation of miR-200c/141 cluster by the PcG protein BMI1. PcG proteins including BMI1 are known to directly bind their target loci [32]. Hence, to determine whether BMI1 directly binds to the *miR-200c/141* promoter region, we performed a chromatin immunoprecipitation linked PCR (ChIP) analysis using chromatin-IP with the BMI1 monoclonal antibody (mAb) and qPCR amplification using 4 different primer sets that cover the promoter region of the miR-200c/141 locus contained in the pGL4.18 vector used in reporter assays. The primer sets were designed to amplify 4 known cis-regulatory transcription factor (TF) binding sites (E-box 2, E-box 3, Z-box 1 and Z-box 2) in the *miR-200c/141* promoter [31, 33]. These TF binding sites are involved in the regulation of *miR-200c/141* promoter by an epithelial-mesenchymal transition (EMT) inducing transcription factor ZEB1 [31]. The results of ChIP analysis indicated significant binding of BMI1 to only region 2, which contained E-box 2 and Z-box 1 TF binding sites (Figure 2B). Although it is not clear whether BMI1 can bind to its target genes independent of PRC1, it was recently reported that it binds to an 89 bp region containing BMI1 response region (BRE) in the p16 promoter via canonical PRC1-independent binding [34]. The exact mechanism of BMI1 binding to region 2, which has very weak homology to p16 BRE (not shown), remains to be explored. It could include binding of BMI1 via its H-T-H-T (helix-turn-helix-turn) or RF (Ring finger) motifs [35], independent of PRC1. Collectively, our results suggested that BMI1 regulates *miR-200c/141* promoter via E-box 2 and Z- box 1 cis-regulatory TF sites present in the region 2 (Figure 2B).

### BMI1 regulates expression of miR-200c/141 *via* E-box 2 and Z-box 1 of the *miR-200c/141* promoter

**Figure 3:**
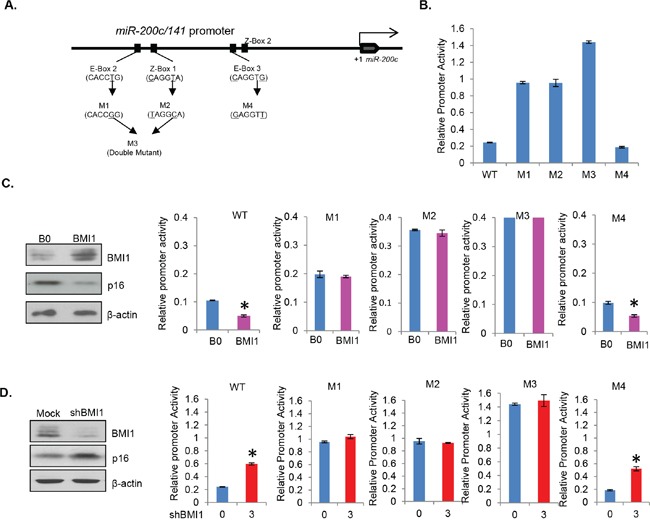
E-box 2 and Z-box 1 are regulatory motifs for miR-200c/141 regulation by BMI1 **A.** Schematic representation of generation of M1, M2, M3 and M4 mutants by site directed mutagenesis. In M1, CACCTG of the E-box 2 was changed to CACCGG, in M2, CAGGTA of the Z-box 1 was changed to TAGGCA, M1 and M2 primers were combined to generate M3 (double mutant). In M4, CAGGTG of the E-box 3 was changed to GAGGTT. **B.** The relative promoter activity of wild type (WT), M1, M2, M3 and M4 *miR-200c/141* promoter-reporter constructs was determined in 293T cells by transient transfection assay as described above in Figure 2. **C.** The relative promoter activity of wild-type and mutant *miR-200c/141* promoter-reporter constructs was determined in stable BMI1 overexpressing cells by transient transfection of reporters. B0 control and BMI1 overexpressing 293T stable cell lines were analyzed for the expression of BMI1, p16 and β-actin by Western blot analysis, and relative promoter activity of WT and M1, M2, M3 and M4 reporter constructs in B0 control and BMI1 cells was determined as described in the Figure 2. **D.** The relative promoter activity of wild-type and mutant *miR-200c/141* promoter-reporter constructs was determined in BMI1 knockdown cells by transient transfection. Mock and BMI1 knockdown cells were generated by transient transfection of the 3 μg of pRS-shBMI1 plasmid. After transfection with the reporter constructs, cells were analyzed for the expression of BMI1, p16 and β-actin by Western blot analysis, and relative promoter activity of WT and M1, M2, M3 and M4 reporter constructs was determined as described in the Figure 2.

The upstream region of *hsa-miR-200c/141* promoter and spacer region between miR-200c and miR-141 genes contain four E-boxes (E-box 1, 2, 3 and 4, GAGGTG) and two Z-boxes (Z-box 1 and 2, CAGGTA) [31]. Since, our cloned promoter region contains four of these putative TF binding motifs, E-box 2 and 3, and Z-box 1 and 2 (Figure 2A), we reasoned that BMI1 possibly regulates miR-200c/141 cluster by binding to one of these motifs. Furthermore, E-Box 2 and Z-Box 1 are located in the sequence region which was amplified with the primer set 2 in ChIP analysis. Therefore, we hypothesized that BMI1 regulates miR-200c/141 expression via these two binding motifs (E-box 2 and Z-box 1). To test this hypothesis, we generated 4 mutants; mutant 1 (M1) in which E-box 2 was mutated, mutant 2 (M2) in which Z-box 1 was mutated, mutant 3 (M3) in which both E-box 2 and Z-box 1 were mutated, and mutant 4 (M4) in which E-box 3 was mutated (Figure 3A). Since BMI1 does not seem to bind to the region 3 of *miR-200c/141* promoter, which was amplified by primer set 3 in ChIP analysis, we expected that BMI1 would not repress it through E-box 3 and therefore used M4 as a negative control.

Next, we performed promoter-reporter assays with the wild type (WT) and mutants (M1-M4) promoter-reporter constructs. Our results showed significantly increased levels of promoter activity in M1, M2 and M3 as compared to the wild-type promoter (Figure 3B). Especially, much higher activity was noted in M3, which has both M1 and M2 mutations (Figure 3B). M4 exhibited much lower activity similar to wild type suggesting that the inhibitory sequences to which BMI1 may bind are intact in M4 and wild type but not in M1, M2 and M3 mutants. These results imply that both E-box 2 and Z-box 1 act as regulatory motifs for transcriptional repression of the miR-200c/141 cluster, and E-box 3, which was mutated in M4 is not a regulatory motif for BMI1-mediated repression of the *miR-200c/141* locus.

Next, to determine whether regulation of the *miR-200c/141* promoter by BMI1 indeed requires E-box 2 and Z-box 1, we tested promoter activity of the WT, M1, M2, M3 and M4 reporter constructs with overexpression or knockdown of BMI1. For BMI1 overexpression, we generated 293T cells stably overexpressing BMI1 using pBabe-BMI1 retrovirus (Figure 3C). The knockdown studies were performed using 293T cells transiently transfected with mock or 3 μg of pRS-BMI1shRNA vector (Figure 3D). The relative promoter activity of WT and all mutants (M1-M4) in 293T-B0 (control) and 293T-BMI1 (BMI1 overexpression) cells was analyzed by the reporter assay. Our results indicated that the promoter activity of WT and M4 mutant constructs is significantly repressed in BMI1 overexpressing cells (Figure 3C), but M1, M2 and M3 did not respond to BMI1 overexpression (Figure 3C). These results suggest that BMI1 regulates *miR-200c/141* promoter via through both E-box 2 and Z-box 1 motifs. In agreement with these results, the WT and M4 promoter-reporter constructs exhibited higher promoter activity in BMI1 knockdown cells (Figure 3D). On the other hand, the promoter activity of M1, M2 and M3 was not affected by the BMI1 knockdown (Figure 3D). These results together with the ChIP analysis indicate that BMI1 regulates *miR-200c/141* promoter via E-box 2 and Z-box 1.

### BMI1 inhibitor PTC-209 upregulates expression of the miR-200c/141 cluster

**Figure 4:**
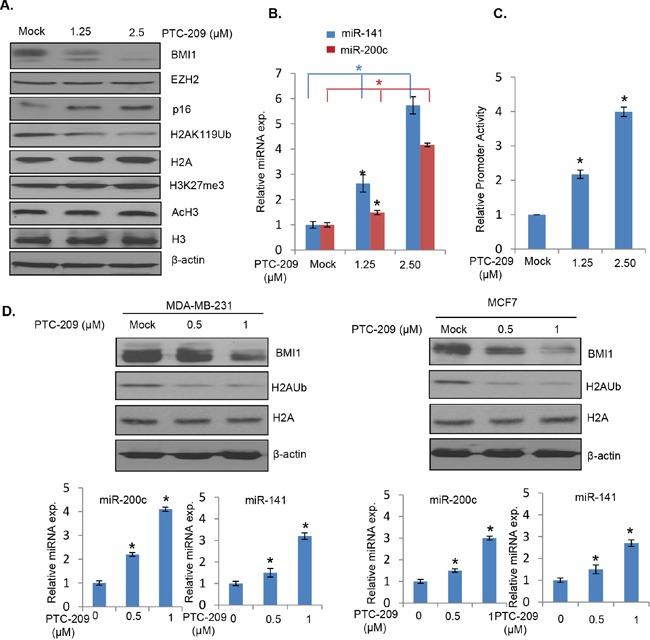
PTC-209 inhibits BMI1 expression, and upregulates miR-200c and miR-141 **A.** 293T cells were mock treated (with DMSO) or treated with the indicated dose of PTC-209 for 48 hours and the expression of BMI1, EZH2, p16, β-actin, H2AK119Ub, total H2A, H3K27me3, AcH3 and total H3 was determined by Western blot analysis. **B.** Expression of miR-200c and miR-141 in mock and PTC-209 treated 293T cells was determined by qRT-PCR analysis. **C.** Luciferase activity of pGL4.8-*PmiR-200c/141* was determined in mock or PTC-209 treated cells as described in the Figure 2. **D.** Western blot analysis of BMI1, H2AK119Ub, total H2A and β-actin in mock or PTC-209 treated MDA-MB-231 (left) and MCF7 (right) cells. In parallel, the mock and PTC-209-treated cells were analyzed for the expression of miR-141 and miR-200c by qRT-PCR analysis (bottom panels). Error bars represent ±S.D, * p <0.05.

Recently, PTC-209 a small molecule inhibitor of BMI1 selected by GMS technology was reported to downregulate BMI1 and colon cancer stem cell (CSC) phenotype in a colorectal cancer model [29]. The PTC-209 does not inhibit BMI1 function by interacting with the protein but rather downregulates *BMI1* transcripts via posttranscriptional mechanism, which is likely to be specific to BMI1. We hypothesized that PTC-209 downregulation of BMI1 may upregulate miR-200c/141 cluster, which could further downregulate BMI1 and oncogenic phenotypes of the cancer cells. To test this hypothesis, we first determined whether PTC-209 treatment indeed results in upregulation of miR-200c and miR-141 by qRT-PCR analysis and promoter-reporter assays. 293T cells were treated with different concentrations of PTC-209 and the expression of BMI1, EZH2, total H2A, H2AK119Ub, total H3, AcH3 and H3K27me3 was determined by the Western blot analysis. In parallel, expression of miR-200c and miR-141 was determined by qRT-PCR analysis. Consistent with the published data in colorectal cells, PTC-209 treatment resulted in specific downregulation of BMI1 and H2AK119Ub (Figure 4A). The expression of EZH2, H3K27me3 and AcH3 was not affected by PTC-209 (Figure 4A). Importantly, PTC-209 treatment resulted in upregulation of both miR-200c and miR-141 (Figure 4B). The results of qRT-PCR were further confirmed by promoter-reporter assays, which showed that the PTC-209 upregulated *miR-200c/141* promoter activity in a dose-dependent manner (Figure 4C). Similar results were obtained with MCF7 and MDA-MB-231 cells upon treatment with PTC-209 (Figure 4D).

**Figure 5:**
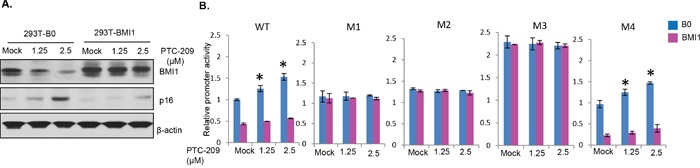
PTC-209 upregulates miR-200c/miR-141 cluster via E-box 2 and Z-box 1 cis-regulatory sites **A.** Control B0 and BMI1 overexpressing 293T cells were mock- or PTC-209- treated for 48 hrs. The total cell lysates were analyzed for the expression of BMI1 and its target p16 by Western blot analysis as described in Figure 1. **B.** Control B0 and BMI1 overexpressing 293T cells were transiently transfected with wild type (WT), M1, M2, M3 and M4 luciferase reporters constructs. The cells were mock- or PTC-209- treated for 48 hrs., and the luciferase activity of the reporters was determined as described in Figure 2.

Next, to determine the mechanism of PTC-209 mediated upregulation of the *miR-200c/141* promoter activity and whether PTC-209 regulation involves E-box 2 and Z-box 1, we performed promoter-reporter assays using transient transfection of wild type and different mutant (M1, M2, M3 and M4) reporters into the B0 control and BMI1 overexpressing 293T cells. The results indicated that PTC-209 had no effect on constitutively expressed BMI1 (Figure 5A). As expected, it only downregulated the endogenous BMI1 (Figure 5A). Accordingly, only wild type and M4 mutant reporters were upregulated in a dose-dependent manner by PTC-209 in B0 cells, presumably due to downregulation of endogenous BMI1 by PTC-209 (Figure 5B). In BMI1 overexpressing cells, the wild type and M4 reporter activity was significantly lower compared to the B0 control cells, and remained lower even after PTC-209 treatment presumably because the exogenously overexpressed BMI1 is not affected by PTC-209 (Figure 5B). The M1, M2 and M3 were not affected in either B0 or BMI1 overexpressing cells (Figure 5B). These mutant reporters exhibited higher activity than wild type and M4 reporters because the potential repressor sites are mutated in M1, M2 and M3 (Figure 5B). Based on these results, we propose that PTC-209 upregulates miR-200c/141 via downregulation of the endogenous BMI1 and that PTC-209 does not directly upregulate miR-200c/141 locus. Collectively, our data suggest that BMI1 inhibition by PTC-209 or its knockdown upregulates expression of miR-200c and miR-141, which further targets BMI1, thereby maintaining expression of both BMI1 and miR-200c/141 cluster via an autoregulatory loop.

### PTC-209 induces premature cellular senescence

**Figure 6:**
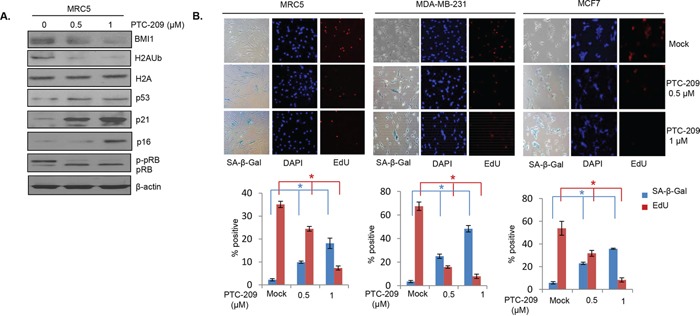
PTC-209 induces premature cellular senescence in human cells **A.** Western blot analysis of BMI1, H2AK119Ub, total H2A and β-actin and molecular markers of senescence (p53, p21, pRB and p16), in MRC5 cells. The cells were mock treated or treated for 48 hrs with PTC-209. **B.** MRC5, MDA-MB-231 and MCF7 cells (as indicated) were treated with PTC-209 for 48 hrs and co-stained with SA-β-gal and EdU to determine the induction of premature senescence by PTC-209. The number of positive cells were counted in 4 random fields and % positive cells for each marker plotted as shown. Error bars represent ±S.D, * p <0.05.

PTC-209 strongly inhibits growth of colon cancer cells and CSC phenotype, and BMI1 downregulation using siRNA approach or its inhibition by histone deacetylase inhibitors (HDACi) and PLK1 inhibitors is known to induce premature senescence in normal and breast cancer cells [27, 28, 36]. Hence, first we determined whether PTC-209 can induce premature cellular senescence in MRC5 fibroblasts. Cells were either mock treated or treated with 0.5 μM and 1 μM of PTC-209 for 48 hrs and stained for senescence associated-beta-galactosidase (SA-β-gal) and EdU (5′ ethynyl -2′-deoxyuridine, a thymidine analog). The stained cells were counted and plotted. We also examined the expression of senescence-associated proteins such as p21, p53, pRB and p16. The results showed that the PTC-209 strongly induced expression of p53, p21 and p16, and increased expression of hypo-phosphorylated pRB in MRC5 cells (Figure 6A). Furthermore, PTC-209 strongly induced premature senescence in these cells as indicated by increase in SA-β-gal positive cells and corresponding decrease in EdU positive cells (Figure 6B). PTC-209 also strongly induced premature senescence in MDA-MB-231 and MCF7 breast cancer cells (Figure 6B middle and right panels). Thus, by inhibiting BMI1, PTC-209 strongly induces premature cellular senescence in normal fibroblasts and breast cancer cells suggesting that PTC-209 also inhibits cancer cell growth via induction of premature senescence, which is a known tumor-suppressive mechanism.

### Inhibition of miR-200c and miR-141 overcomes PTC-209 inhibitory effects on oncogenic phenotypes

**Figure 7:**
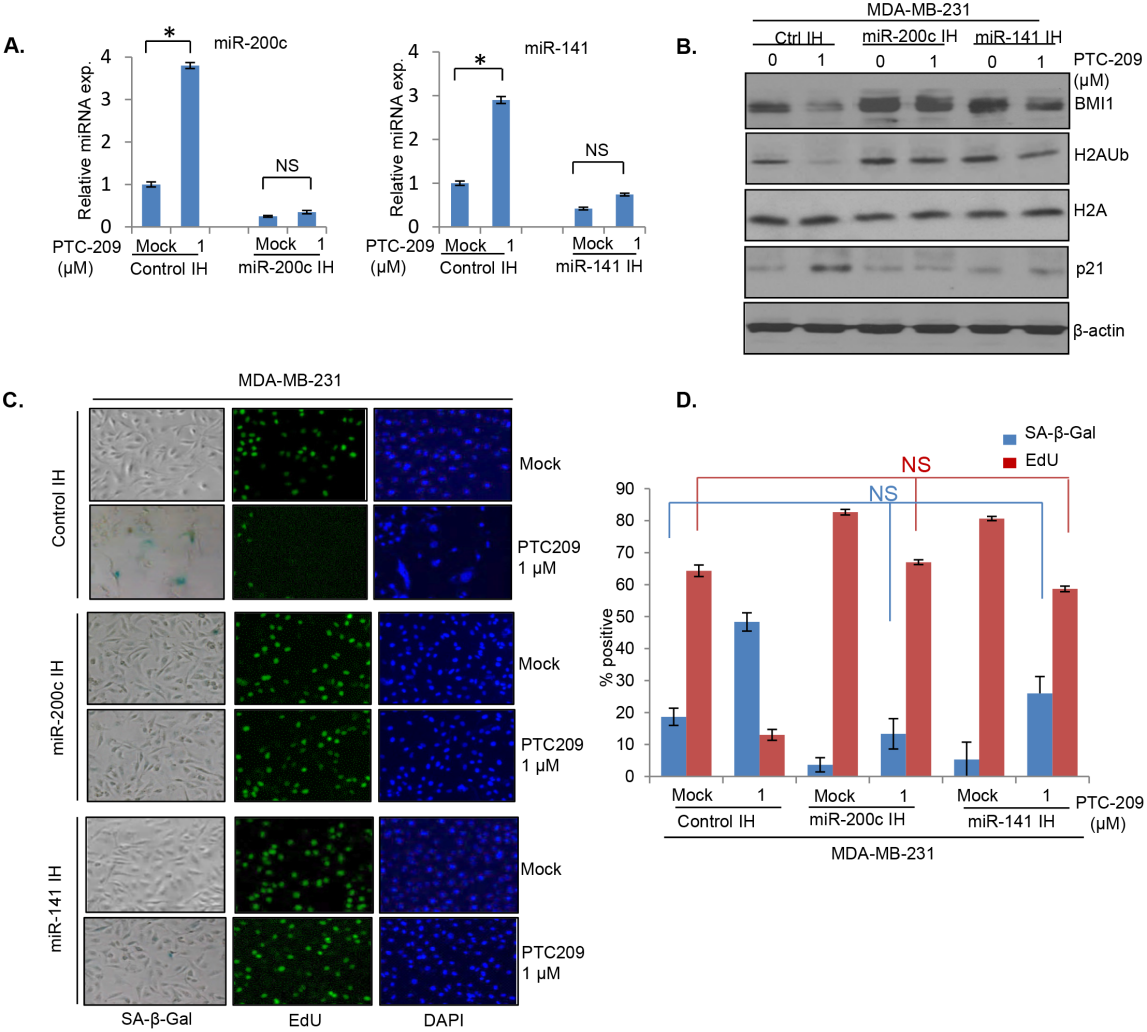
Inhibitors of miR-141 and miR-200c overcome induction of premature senescence by PTC-209 in breast cancer cells **A.** qRT-PCR analysis of miR-141 and miR-200c in control, miR-141 inhibitor and miR-200c inhibitor expressing cells. **B.** Western blot analysis of BMI1, H2AK119Ub, total H2A, p21 and β-actin in control, and miR-141 and miR-200c inhibitors expressing MDA-MB-231 cells treated with DMSO (mock) or PTC-209 (1.0 μM) for 48 hrs. **C.** SA-β-gal/EdU co-staining of control, and miR-141 and miR-200c inhibitors expressing MDA-MB-231 cells either mock or PTC-209 treated as described in the Methods. **D.** The percent positive SA-β-gal and EdU stained cells were counted and plotted. Error bars represent ±S.D, * p <0.05, NS- not significant.

Next, to determine the functional significance of the miR-200c/141-BMI1 autoregulatory loop, we examined whether PTC-209 inhibits oncogenic activity of BMI1 via upregulation of miR-200c/141 cluster. First, we determined whether knockdown of miR-200c and miR-141 can overcome the induction of premature senescence by PTC-209 in MDA-MB-231 and MCF7 cells. We generated MDA-MB-231 and MCF7 cells expressing a control vector and inhibitors of miR-200c and miR-141 designated as control IH, miR-200c IH and miR-141 IH breast cancer cells respectively. Next, cells were either mock treated or treated with 1.0 μm PTC-209 for 48 hrs, and then examined for the expression of miR-200c and miR-141. The results indicated that PTC-209 treatment led to induction of miR-200c and miR-141 in control IH but not in miR-200c IH and miR-141 IH breast cancer cells (Figure 7A). We also analyzed expression of BMI1, H2AUb, H2A and p21 using Western blot analysis. Our data indicated that compared to control IH cells, PTC-209 did not significantly downregulate BMI1 and H2AUb in miR-200c IH and miR-141 IH cells (Figure 7B). Because these cells are p16 negative, we only determined the expression of p21 as a senescence effector in this context. The results showed that the PTC-209 strongly induced p21 in control IH cells and that inhibition of either miR-200c or miR-141 could suppress p21 induction by PTC-209 (Figure 7B). Next, mock- and PTC-209- treated cells were plated for SA-β-gal and EdU co-staining. The percent positives for each marker were quantified and plotted. The results indicated that the PTC-209 treatment led to the induction of premature senescence in control IH cells but not in cells expressing inhibitors of either miR-200c or miR-141 (Figure 7C, 7D). Similar results were obtained in MCF7 cells (not shown).

**Figure 8:**
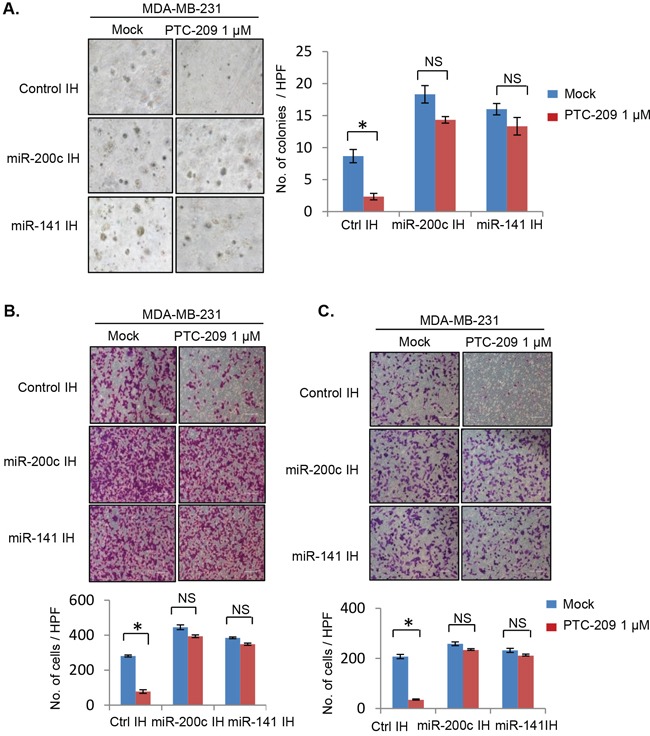
PTC-209 inhibits oncogenic phenotypes in MDA-MB-231cells and inhibitors of miR-200c and 141 overcome PTC-209 inhibitory effects **A.** Soft-agar colony formation assay, **B.** Migration and **C.** Invasion assay of control, and miR-141 and miR-200c inhibitors expressing MDA-MB-231 cells treated with DMSO (mock) or PTC-209 (1.0 μM) for 7 days (soft agar), or 48 hrs (migration and invasion assays). The number of colonies (soft agar), migrated and cells that invaded through Matrigel were quantified and plotted as described in the Methods. Error bars represent ±S.D, * p <0.05, NS- not significant.

We further studied whether PTC-209 inhibits soft agar colony formation, migration and invasion in MDA-MB-231 cells, and whether miR-141 and miR-200c inhibitors can overcome the inhibitory effect of PTC-209 on these oncogenic phenotypes (Figure 8). The results of the soft-agar colony formation suggest that PTC-209 significantly inhibited soft agar colony formation in control IH but not in miR-200c IH and miR-141 IH cells (Figure 8A). Similarly, results of the migration and invasion assays showed that inhibition of either miR-200c or miR-141 can overcome PTC-209 mediated inhibition of migration and invasion in MDA-MB-231 cells (Figure 8B, 8C).

**Figure 9:**
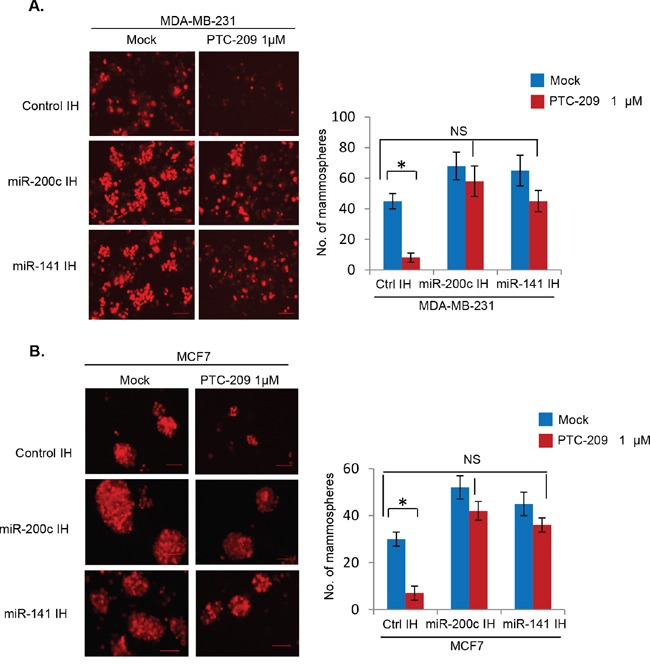
PTC-209 inhibits mammosphere formation, and inhibition of miR-141 and miR-200c overcomes PTC-209 inhibitory activity on mammosphere formation in breast cancer cells The mammosphere formation assay using control, and miR-141 and miR-200c inhibitors expressing MDA-MB-231 or MCF7 cells treated with DMSO (mock) or PTC-209 (1.0 μM) (as indicated) was performed as described in the Methods. The number of mammospheres (>50 μm) were quantified after 5 days of plating, and plotted. Error bars represent ±S.D, * p <0.05, NS- not significant.

The PTC-209 exhibits remarkable inhibitory activity towards colon CSCs [29, 37]. Hence, we examined whether PTC-209 similarly inhibits breast CSC phenotype using mammosphere and Aldefluor assays. The control IH, miR-200c IH and miR-141 IH breast cancer cells were plated for mammosphere formation, and treated with PTC-209. After 5 days the numbers and sizes of mammospheres were counted, photographed and plotted (Figure 9A, 9B). The results of mammosphere formation assay indicated that PTC-209 strongly inhibits mammosphere formation in control MDA-MB-231 and MCF7 cells, and that downregulation of either miR-141 or miR-200c using respective inhibitors is sufficient to overcome inhibition of mammosphere formation by PTC-209 (Figure 9A, 9B).

**Figure 10:**
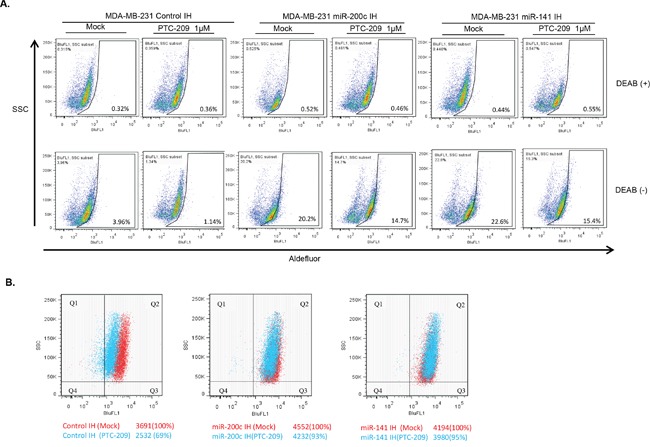
PTC-209 inhibits BCSC markers and inhibition of miR-141 and miR-200c overcome PTC-209 inhibitory effect on BCSC markers **A.** ALDH1 activity of control, and miR-141 and miR-200c inhibitors expressing MDA-MB-231 cells treated with DMSO (mock) or PTC-209 (1.0 μM) (as indicated) was determined using an ALDEFLUOR kit as described in the Experimental Methods. DEAB+ sets are controls. **B.** CD44 expression in control, miR-141 inhibitor and miR-200c inhibitor expressing MDA-MB-231 cells treated with DMSO (mock) or PTC-209 (1.0 μM) (as indicated) was determined by FACS analysis described in the Methods.

Next, we carried out the Aldefluor assay to analyze aldehyde dehydrogenase isoform 1 (ALDH1) activity, which is a known marker of the breast CSCs [38]. The control IH, miR-200c IH and miR-141 IH breast cancer cells were plated, treated with 1 μM PTC-209 for 48 hrs and ALDH1 activity was determined using the ALDEFLUOR assay kit (Stem Cell Technologies, Vancouver, BC, Canada). The results indicated that PTC-209 treatment led to 84% decrease of ALDH1 activity in control MDA-MB-231 cells, while miR-141 IH and miR-200c IH cells exhibited 30% and 28% decrease respectively with PTC-209 treatment (Figure 10A). The miR-141 IH and miR-200c IH cells exhibit higher ALDH1 activity compared to control IH, confirming the negative regulatory role of miR-141 and miR-200c in breast CSC phenotype. Similar results were obtained with MCF7 cells (not shown). Thus, our data indicate that inhibition of miR-141 and miR-200c partially reversed the decrease in ALDH1 activity caused by the PTC-209 treatment.

We also analyzed expression of CD44, a known marker of breast CSCs by FACS analysis of PTC-209 treated control and miR-200c or miR-141 inhibitor expressing cells. The data indicated that PTC-209 treatment for 48 hrs led to a 40% decrease in CD44 expression in control cells, while in miR-141 IH and miR-200c IH cells the decrease in CD44 expression was only 4% and 7% respectively (Figure 10B). These data indicate that inhibition of either miR-200c or miR-141 can overcome the decrease in fraction of CD44 expressing cells by PTC-209 treatment. Collectively, our data indicate that PTC-209 induced inhibition of CSC phenotype is mediated by upregulation of miR-200 family members miR-200c and miR-141, and that the inhibition of either of these family members can overcome the inhibitory effect on oncogenic phenotypes by PTC-209 in breast cancer cells.

## DISCUSSION

PcG protein BMI1 is an important regulator of proliferation and cell senescence [22, 23]. It also regulates oncogenic phenotypes such as migration, invasion and metastasis of cancer cells [17, 18, 22, 39, 40]. More importantly, BMI1 promotes CSC properties and therapy resistance in breast, prostate, lung and colorectal cancers [20, 41]. Therefore, therapeutic targeting of BMI1 could help in the treatment of breast, prostate, colorectal and possibly other cancers, and overcome the disease recurrence. BMI1, EZH2 and other PcG proteins can be targeted by pharmacological inhibitors of HDACs [24, 42], which are known for tumor suppressive properties. More recently, PTC-209, a small molecule inhibitor of BMI1 was shown to target self-renewal of colon CSCs and inhibit colorectal cancer [29, 43]. While targeting of self-renewal of colon CSCs by PTC-209 is likely to be due to the downregulation of BMI1, it is not clear how mechanistically PTC-209 downregulates BMI1. Since, the reporter used in the identification and characterization of PTC-209 contained 5′ and 3′ untranslated region (UTR) of the *BMI1* gene (29), it is possible that PTC-209 regulates BMI1 via miRNAs that target 3′UTR of the *BMI1* gene. It is also possible that PTC-209 directly upregulates miRNAs that inhibit CSC phenotype. Hence, here we focused on miR-200c and miR-141, which are known to posttranscriptionally target BMI1 and its oncogenic activity including regulation of CSC phenotype [27, 28, 41]. Because PTC-209 exhibits strong tumor suppressive activity, it is likely that it also regulates cellular senescence, which is a natural tumor suppressor mechanism [44]. Indeed, our data demonstrate that PTC-209 strongly induces premature senescence not only in normal human diploid fibroblasts but also in breast cancer cells. Previously, we have shown that both miR-141 and miR-200c are important regulators of cell senescence (27,28). Although it is possible that these miRNAs can induce senescence via regulation of other molecular targets, BMI1 is an established regulator of senescence, hence, it is likely that PTC-209 induces senescence by perturbing the miR-200c/141-BMI1 autoregulatory loop. Indeed, our results show that by inhibiting miR-200c or miR-141, the senescence inducing activity and other tumor suppressive activities of PTC-209 can be abrogated in breast cancer cells.

**Figure 11:**
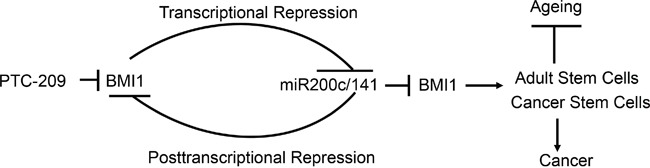
Schematic representation of the miR-200c/141-BMI1 autoregulatory loop and its relevance to cancer and ageing PTC-209 inhibits BMI1 expression. BMI1 transcriptionally downregulates tumor suppressive miRNAs, in particular miR-141 and miR-200c. MiR-141 and miR-200c posttranscriptionally downregulate BMI1, which in turn transcriptionally downregulates expression of miR-200c/141 cluster completing an autoregulatory loop. A fine-tuned level of BMI1 and miR-200c/141 via this autoregulatory loop maintains a normal balance between the proliferation and senescence in an organism necessary for its normal physiology. Upregulation of BMI1 due to the inhibition of miR-200c/141 cluster could promote cancer stem cell phenotype and therefore development of cancer, while upregulation of miR-200c/141 cluster could inhibit adult- or organ-specific stem cells, which may promote age-related pathologies and premature ageing.

ZEB1 and miR-200c/141 regulate each other via a negative feedback loop or reciprocal repression [31]. We have recently shown that a similar autoregulatory loop regulates expression of the PcG proteins and miR-31, another tumor suppressive miRNA [26]. Here, we propose a similar model to regulate expression of miR-200c/141 and BMI1, and physiological function of BMI1 (Figure 11). Although, here we mainly focused on the physiological function of BMI1 in the context of the CSC regulation, BMI1 is a known regulator of normal and adult stem cells [45]. It's deficiency causes depletion of neural, hematopoietic and other adult organ-specific stem cells in the mouse model, and BMI1 knockout mice exhibit segmental features of the accelerated ageing and other pathologies such as type II diabetes [23, 43, 46–48]. Hence, it is likely that miR-200c/141-BMI1 autoregulatory loop is highly relevant to the ageing process (Figure 11). In summary, our studies suggest that BMI1 autoregulates its expression via repression of miR-200c/141 locus, and BMI1 inhibitory reagents such as PTC-209 and possibly HDACi inhibit BMI1 expression via upregulation of miR-200c/141 cluster, which can further downregulate BMI1 by posttranscriptional mechanism. BMI1 is an important physiological regulator [43], as its deregulation is associated with cancer, and adult stem cell depletion and accelerated ageing. We speculate that this autoregulatory loop maintains a basal endogenous level expression of BMI1 and miR-200c/141 necessary for the proper physiology of an organism, and that its deregulation can result in pathological conditions such as cancer and ageing.

## MATERIALS AND METHODS

### Cell culture and reagents

MDA-MB-231, MCF7 and 293T (SV40 T expressing HEK293) cells were obtained from American Type Culture Collection (ATCC) (Manassas, VA). The cells were cultured in Dulbecco's Modified Eagle's Medium (DMEM) containing 5% Fetal Bovine Serum (FBS) as described [25, 27]. MRC5 strain of human diploid fibroblast (HDF) was obtained from the Coriell Institute for Medical Research Aging Cell Repository (Camden, NJ), and cultured as described (25, 27). PTC-209 (N-(2,6-dibromo-4-methoxyphenyl)-4-(2-methylimidazo[1,2-a]pyrimidin-3-yl)-2-thiazolamine) was obtained from the Cayman Chemicals (Ann Arbor, MI), dissolved in DMSO (dimethyl sulfoxide), and added to the cell culture medium as described [24].

### Expression vectors, promoter-reporters, transient transfections, retrovirus and lentivirus production, and luciferase assays

Lentiviral vector pEZX-MR03 expressing miR-200c and miR-141 pre-miRNAs, and pEZX-AM03 expressing miR-200c and miR-141 inhibitors (miArrest™ miRNA) were obtained from the Genecopoeia (Rockville, MD). The pBabe-puro retroviral vector overexpressing wild type BMI1, and BMI1 shRNA (in pRetro-super (RS) vector) and pcDNA-BMI1 have been described previously [25, 27]. For stable clone generation, retroviruses were produced, and target cells were infected and selected in 0.5-1 μg/ml puromycin as described [25, 27, 49]. Cloning of the promoter region of the miR-200c/141 cluster has been described earlier [28]. Briefly, to generate a wild-type *hsa-miR-200c/141* promoter reporter construct, the upstream promoter region (−683 to −44 bp) was PCR-amplified with a primer set (WT F and R primers, Table 1) and cloned into the pGL4.18 vector (Promega, Madison, WI). The mutant reporter constructs were generated using Quick Change Site-Directed Mutagenesis Kit (Agilent Technology) according to the manufacturer's protocol. The primer sets used in the site-directed mutagenesis are described in Table 1. The primer sets M1 and M2 were used to generate mutant 3 (M3). The reporter constructs were transiently transfected into 293T cells using calcium phosphate method, and promoter-reporter assays were performed using Dual-Luciferase® Reporter Assay System as described [25, 27].

**Table 1:** Sets of primers used to generate miR-200c/141 promoter luciferase constructs

Primer		Primer Sequence
WT	F	5′-GACTCGAGAGGGGTGAGACTAGGCAGGT-3′
	R	5′-CCAAGCTTAGGATCCCTGCGGAAAAG-3′
M1 *	F	5′-CACCCTGCGCACCGGTGTGTCCTCC-3′
	R	5′-GGAGGACACACCGGTGCGCAGGGTG-3′
M2 *	F	5′-CGCAGGCTCCTTGCCTAGGCCCCGCCTGC-3′
	R	5′-GCAGGCGGGGCCTAGGCAAGGAGCCTGCG-3′
M4 *	F	5′-GTCACCGGGCCAACCTCCCCCCAGAGAG-3′
	R	5′-CTCTCTGGGGGGAGGTTGGCCCGGTGAC-3′

* indicates primer sets used for site-directed mutagenesis.

### Antibodies and western blot analyses

The BMI1 mouse monoclonal antibody (mAb) F6, p16INK4a (p16) mAb and β-actin mAb were obtained from Invitrogen (Carlsbad, CA), Santa Cruz Biotechnology (Santa Cruz, CA) and Sigma-Aldrich (St Louis, MO), respectively. The rabbit polyclonal antibodies (pAbs) against H3 and H2A, and mAbs against H4, H3 and acetyl histone H3 were obtained from Cell Signaling Technology (Danvers, MA). The H3K27me3 and H2AK119Ub pAbs were obtained from Millipore (Billerica, MA). Western blot analyses using antibodies specific to the protein of interest were done as described previously [24, 50].

### Quantitative RT-PCR (qRT-PCR) and chromatin-immunoprecipitation-linked PCR (ChIP) analyses

The qRT-PCR assays were performed as described [27]. Briefly, total RNA was isolated using TRIzol reagent as described by manufacturer (Invitrogen, Carlsbad, CA), and treated with DNase (Promega, Madison, WI). For miRNA qRT-PCR, miR-141 and miR-200c specific primers, and cDNA synthesis kit were from Quanta Biosciences (Gaithersburg, MD). The PCR conditions consisted of an initial activation at 50°C for 2 min, 95°C for 20 sec followed by 40 cycles of 95°C for 1 sec, and 60°C for 20 sec in the Step One Plus Real-Time PCR system (Applied Biosystems). The Ct (threshold cycle) value of each primer was normalized to that of RNU6B for miRNA or GAPDH for other genes as internal controls. The chromatin immunoprecipitation (ChIP) assays were performed as described [25, 50]. The immunoprecipitated chromatin was amplified using different sets of *miR-200c/141* promoter specific primers (Table 2) by qPCR in a Step-one plus RT-PCR machine (ABI).

**Table 2:** ChIP assay primers

Primer	Location		Primer Sequence
1	(−543 to −683)	F	5′-AGGGGTGAGACTAGGCAGGTTGG-3′
		R	5′-ATAGGCTGCCCCCCCACTG-3′
2	(−370 to −546)	F	5′-TATGGCAGGAGGACACACCTGTG-3′
		R	5′-CCCCAGAGAGTGGGTGCTGG-3′
3	(−170 to −372)	F	5′-GGGCAGGTGGGCCCGGTGA-3′
		R	5′-CCAGGTTGCAGTCCAAGCA-3′
4	(0 to -172)	F	5′-GGGCCTCGTGATCAGCGAC-3′
		R	5′-CACCTTGGGTCAGGCAGCTT-3′

### Proliferation, senescence and mammosphere formation assays, and analysis of cancer stem cell (CSC) markers

Proliferation and senescence assays were performed as described [36, 51]. The EdU (5′ ethynyl -2′-deoxyuridine, a thymidine analog) and senescence-associated beta galactosidase (SA-β-Gal) co-staining was performed as described [52]. Images were captured with a Nikon Eclipse Ti microscope camera under 10X magnification and stained cells were counted as described [53]. Mammosphere formation and Aldefluor assays, and FACS analysis of CD44 marker to determine breast cancer stem cell phenotype were done as described [25, 26, 28].

### Statistical analysis

All experiments were performed multiple times with triplicates for each group. Results are presented as the mean ± S.D., and the statistical significance was determined using Student's *t* test. The *p* < 0.05 was considered significant.

## References

[1] Bartel DP (2004). MicroRNAs: genomics, biogenesis, mechanism, and function. Cell.

[2] Bartel DP (2009). MicroRNAs: target recognition and regulatory functions. Cell.

[3] Croce CM (2009). Causes and consequences of microRNA dysregulation in cancer. Nature reviews Genetics.

[4] Garofalo M, Condorelli G, Croce CM (2008). MicroRNAs in diseases and drug response. Current opinion in pharmacology.

[5] Garzon R, Calin GA, Croce CM (2009). MicroRNAs in Cancer. Annual review of medicine.

[6] Iorio MV, Croce CM (2012). microRNA involvement in human cancer. Carcinogenesis.

[7] Dvinge H, Git A, Graf S, Salmon-Divon M, Curtis C, Sottoriva A, Zhao Y, Hirst M, Armisen J, Miska EA, Chin SF, Provenzano E, Turashvili G (2013). The shaping and functional consequences of the microRNA landscape in breast cancer. Nature.

[8] O'Day E, Lal A (2010). MicroRNAs and their target gene networks in breast cancer. Breast cancer research.

[9] Feng X, Wang Z, Fillmore R, Xi Y (2014). MiR-200, a new star miRNA in human cancer. Cancer letters.

[10] Gregory PA, Bert AG, Paterson EL, Barry SC, Tsykin A, Farshid G, Vadas MA, Khew-Goodall Y, Goodall GJ (2008). The miR-200 family and miR-205 regulate epithelial to mesenchymal transition by targeting ZEB1 and SIP1. Nature cell biology.

[11] Howe EN, Cochrane DR, Richer JK (2011). Targets of miR-200c mediate suppression of cell motility and anoikis resistance. Breast cancer research.

[12] Di Croce L, Helin K (2013). Transcriptional regulation by Polycomb group proteins. Nature structural & molecular biology.

[13] Simon JA, Kingston RE (2013). Occupying chromatin: Polycomb mechanisms for getting to genomic targets, stopping transcriptional traffic, and staying put. Molecular cell.

[14] Glinsky GV, Berezovska O, Glinskii AB (2005). Microarray analysis identifies a death-from-cancer signature predicting therapy failure in patients with multiple types of cancer. The Journal of clinical investigation.

[15] Guo WJ, Zeng MS, Yadav A, Song LB, Guo BH, Band V, Dimri GP (2007). Mel-18 acts as a tumor suppressor by repressing Bmi-1 expression and down-regulating Akt activity in breast cancer cells. Cancer research.

[16] Kim JH, Yoon SY, Jeong SH, Kim SY, Moon SK, Joo JH, Lee Y, Choe IS, Kim JW (2004). Overexpression of Bmi-1 oncoprotein correlates with axillary lymph node metastases in invasive ductal breast cancer. Breast.

[17] Datta S, Hoenerhoff MJ, Bommi P, Sainger R, Guo WJ, Dimri M, Band H, Band V, Green JE, Dimri GP (2007). Bmi-1 cooperates with H-Ras to transform human mammary epithelial cells via dysregulation of multiple growth-regulatory pathways. Cancer research.

[18] Hoenerhoff MJ, Chu I, Barkan D, Liu ZY, Datta S, Dimri GP, Green JE (2009). BMI1 cooperates with H-RAS to induce an aggressive breast cancer phenotype with brain metastases. Oncogene.

[19] Kleer CG, Cao Q, Varambally S, Shen R, Ota I, Tomlins SA, Ghosh D, Sewalt RG, Otte AP, Hayes DF, Sabel MS, Livant D, Weiss SJ, Rubin MA, Chinnaiyan AM (2003). EZH2 is a marker of aggressive breast cancer and promotes neoplastic transformation of breast epithelial cells. Proc Natl Acad Sci U S A.

[20] Lukacs RU, Memarzadeh S, Wu H, Witte ON (2010). Bmi-1 is a crucial regulator of prostate stem cell self-renewal and malignant transformation. Cell stem cell.

[21] Siddique HR, Saleem M (2012). Role of BMI1, a stem cell factor, in cancer recurrence and chemoresistance: preclinical and clinical evidences. Stem cells.

[22] Itahana K, Zou Y, Itahana Y, Martinez JL, Beausejour C, Jacobs JJ, Van Lohuizen M, Band V, Campisi J, Dimri GP (2003). Control of the replicative life span of human fibroblasts by p16 and the polycomb protein Bmi-1. Molecular and cellular biology.

[23] Jacobs JJ, Kieboom K, Marino S, DePinho RA, van Lohuizen M (1999). The oncogene and Polycomb-group gene bmi-1 regulates cell proliferation and senescence through the ink4a locus. Nature.

[24] Bommi PV, Dimri M, Sahasrabuddhe AA, Khandekar J, Dimri GP (2010). The polycomb group protein BMI1 is a transcriptional target of HDAC inhibitors. Cell cycle.

[25] Cho JH, Dimri M, Dimri GP (2013). A positive feedback loop regulates the expression of polycomb group protein BMI1 via WNT signaling pathway. The Journal of biological chemistry.

[26] Cho JH, Dimri M, Dimri GP (2015). MicroRNA-31 is a transcriptional target of histone deacetylase inhibitors and a regulator of cellular senescence. The Journal of biological chemistry.

[27] Dimri M, Carroll JD, Cho JH, Dimri GP (2013). microRNA-141 regulates BMI1 expression and induces senescence in human diploid fibroblasts. Cell cycle.

[28] Dimri M, Cho JH, Kang M, Dimri GP (2015). PLK1 Inhibition Down-regulates Polycomb Group Protein BMI1 via Modulation of the miR-200c/141 Cluster. The Journal of biological chemistry.

[29] Kreso A, van Galen P, Pedley NM, Lima-Fernandes E, Frelin C, Davis T, Cao L, Baiazitov R, Du W, Sydorenko N, Moon YC, Gibson L, Wang Y (2014). Self-renewal as a therapeutic target in human colorectal cancer. Nature medicine.

[30] Yamagishi M, Nakano K, Miyake A, Yamochi T, Kagami Y, Tsutsumi A, Matsuda Y, Sato-Otsubo A, Muto S, Utsunomiya A, Yamaguchi K, Uchimaru K (2012). Polycomb-mediated loss of miR-31 activates NIK-dependent NF-kappaB pathway in adult T cell leukemia and other cancers. Cancer cell.

[31] Burk U, Schubert J, Wellner U, Schmalhofer O, Vincan E, Spaderna S, Brabletz T (2008). A reciprocal repression between ZEB1 and members of the miR-200 family promotes EMT and invasion in cancer cells. EMBO reports.

[32] Bracken AP, Kleine-Kohlbrecher D, Dietrich N, Pasini D, Gargiulo G, Beekman C, Theilgaard-Monch K, Minucci S, Porse BT, Marine JC, Hansen KH, Helin K (2007). The Polycomb group proteins bind throughout the INK4A-ARF locus and are disassociated in senescent cells. Genes & development.

[33] Neves R, Scheel C, Weinhold S, Honisch E, Iwaniuk KM, Trompeter HI, Niederacher D, Wernet P, Santourlidis S, Uhrberg M (2010). Role of DNA methylation in miR-200c/141 cluster silencing in invasive breast cancer cells. BMC research notes.

[34] Meng S, Luo M, Sun H, Yu X, Shen M, Zhang Q, Zhou R, Ju X, Tao W, Liu D, Deng H, Lu Z (2010). Identification and characterization of Bmi-1-responding element within the human p16 promoter. The Journal of biological chemistry.

[35] Yadav AK, Sahasrabuddhe AA, Dimri M, Bommi PV, Sainger R, Dimri GP (2010). Deletion analysis of BMI1 oncoprotein identifies its negative regulatory domain. Molecular cancer.

[36] Guo WJ, Datta S, Band V, Dimri GP (2007). Mel-18, a polycomb group protein, regulates cell proliferation and senescence via transcriptional repression of Bmi-1 and c-Myc oncoproteins. Molecular biology of the cell.

[37] Wicha MS (2014). Targeting self-renewal, an Achilles' heel of cancer stem cells. Nature medicine.

[38] Ginestier C, Hur MH, Charafe-Jauffret E, Monville F, Dutcher J, Brown M, Jacquemier J, Viens P, Kleer CG, Liu S, Schott A, Hayes D, Birnbaum D (2007). ALDH1 is a marker of normal and malignant human mammary stem cells and a predictor of poor clinical outcome. Cell stem cell.

[39] Dimri GP, Martinez JL, Jacobs JJ, Keblusek P, Itahana K, Van Lohuizen M, Campisi J, Wazer DE, Band V (2002). The Bmi-1 oncogene induces telomerase activity and immortalizes human mammary epithelial cells. Cancer research.

[40] Kim RH, Lieberman MB, Lee R, Shin KH, Mehrazarin S, Oh JE, Park NH, Kang MK (2010). Bmi-1 extends the life span of normal human oral keratinocytes by inhibiting the TGF-beta signaling. Experimental cell research.

[41] Shimono Y, Zabala M, Cho RW, Lobo N, Dalerba P, Qian D, Diehn M, Liu H, Panula SP, Chiao E, Dirbas FM, Somlo G, Pera RA (2009). Downregulation of miRNA-200c links breast cancer stem cells with normal stem cells. Cell.

[42] Fiskus W, Pranpat M, Balasis M, Herger B, Rao R, Chinnaiyan A, Atadja P, Bhalla K (2006). Histone deacetylase inhibitors deplete enhancer of zeste 2 and associated polycomb repressive complex 2 proteins in human acute leukemia cells. Molecular cancer therapeutics.

[43] Bhattacharya R, Mustafi SB, Street M, Dey A, Dwivedi SK (2015). Bmi-1: At the crossroads of physiological and pathological biology. Genes & Diseases.

[44] Dimri GP (2005). What has senescence got to do with cancer?. Cancer cell.

[45] Park IK, Morrison SJ, Clarke MF (2004). Bmi1, stem cells, and senescence regulation. The Journal of clinical investigation.

[46] Chatoo W, Abdouh M, David J, Champagne MP, Ferreira J, Rodier F, Bernier G (2009). The polycomb group gene Bmi1 regulates antioxidant defenses in neurons by repressing p53 pro-oxidant activity. The Journal of neuroscience.

[47] Dhawan S, Tschen SI, Bhushan A (2009). Bmi-1 regulates the Ink4a/Arf locus to control pancreatic beta-cell proliferation. Genes & development.

[48] Gu M, Shen L, Bai L, Gao J, Marshall C, Wu T, Ding J, Miao D, Xiao M (2014). Heterozygous knockout of the Bmi-1 gene causes an early onset of phenotypes associated with brain aging. Age.

[49] Dimri GP, Itahana K, Acosta M, Campisi J (2000). Regulation of a senescence checkpoint response by the E2F1 transcription factor and p14(ARF) tumor suppressor. Molecular and cellular biology.

[50] Sahasrabuddhe AA, Dimri M, Bommi PV, Dimri GP (2011). betaTrCP regulates BMI1 protein turnover via ubiquitination and degradation. Cell cycle.

[51] Dimri GP, Lee X, Basile G, Acosta M, Scott G, Roskelley C, Medrano EE, Linskens M, Rubelj I, Pereira-Smith O, Peacocke M, Campisi J (1995). A biomarker that identifies senescent human cells in culture and in aging skin in vivo. Proceedings of the National Academy of Sciences of the United States of America.

[52] Itahana K, Itahana Y, Dimri GP (2013). Colorimetric detection of senescence-associated beta galactosidase. Methods in molecular biology.

[53] Dimri M, Naramura M, Duan L, Chen J, Ortega-Cava C, Chen G, Goswami R, Fernandes N, Gao Q, Dimri GP, Band V, Band H (2007). Modeling breast cancer-associated c-Src and EGFR overexpression in human MECs: c-Src and EGFR cooperatively promote aberrant three-dimensional acinar structure and invasive behavior. Cancer research.

